# Integrated Transcriptomics and Metabolomics to Reveal the Regulatory Mechanisms of Flavonoid Biosynthesis in *Persicaria capitata* Under Different Nitrogen Fertilization Levels

**DOI:** 10.3390/metabo16090616

**Published:** 2026-08-27

**Authors:** Jiangyong An, Shaoyan Zhang, Yuxin Wang, Fuqiang Du, Tao Zhang

**Affiliations:** 1 School of Pharmacy, Guizhou University of Traditional Chinese Medicine, Guiyang 550025, China; 18798142620@163.com (S.Z.); 18766119298@163.com (Y.W.); zhangtao021@gzy.edu.cn (T.Z.); 2 Guizhou Warmen Pharmaceutical Co., Ltd., Guiyang 550018, China; nihaodfq@163.com

**Keywords:** *Persicaria capitata*, nitrogen fertilizer level, secondary metabolites, flavonoid metabolites

## Abstract

Background: *Persicaria capitata* is a perennial herb and one of the top ten authentic Miao medicinal plants in Guizhou province, China, with flavonoids serving as its primary bioactive components. Agricultural production practices have shown that nitrogen fertilizer application can reduce flavonoid accumulation in *P. capitata*, although the underlying molecular regulatory mechanism remains unclear. Methods: In this study, a pot experiment was performed to investigate the effects of different nitrogen treatments on the growth performance and yield of *P. capitata*. Moreover, the related changes in transcriptomic characteristics and metabolic profiles were also systematically analyzed. Results: Nitrogen fertilization significantly promoted stem elongation, branch number, and yield formation. The maximum stem length, branch number, and single-plant yield were recorded in the 0.3 g N/kg treatment group as reaching 80.74 cm, 37.30, and 123.64 g/plant, respectively. Transcriptomic screening yielded 10,880 differentially expressed genes (DEGs), and the number of downregulated DEGs was consistently higher than upregulated DEGs across all comparison groups. Metabolomic analysis identified a total of 3356 differentially accumulated metabolites, including 168 differential flavonoid metabolites. With the increase in nitrogen application rate, most flavonoid compounds, such as chlorogenic acid, 5-hydroxyferulic acid, and 4-hydroxycinnamic acid, exhibited a significantly downregulated accumulation pattern. An integrated transcriptomic and metabolomic analysis revealed that the differential flavonoid metabolites and functional genes were predominantly enriched in the phenylpropanoid biosynthesis, flavonoid biosynthesis, and flavone and flavonol metabolism pathways. The decreased expression of key structural genes, including *PAL*, *4CL*, *CHS*, *FLS*, *F3H*, and *F3′H*, inhibited the biosynthesis and accumulation of core flavonoid components (e.g., chlorogenic acid, naringenin, and luteolin), thereby deteriorating the medicinal quality of *P. capitata*. Conclusions: Nitrogen supplementation increases the yield of *P. capitata* but inhibits flavonoid biosynthesis. Downregulated expression of key genes in flavonoid metabolism is responsible for the decreased flavonoid content. This study provides theoretical support for optimized nitrogen management to coordinate yield formation and quality improvement in planting practices for *P. capitata*.

## 1. Introduction

*Persicaria capitata*, commonly known as “Touhualiao”, is a distinctive Miao ethnic medicine. This species is primarily distributed across the provinces of Guizhou and Yunnan in southwestern China [1]. Its dried whole plant or aerial parts are used in clinical practice, and it has been recorded in the “Guizhou Provincial Standards for Chinese and Ethnic Medicinal Materials”. Pharmacologically, this herb exerts multiple therapeutic effects, including heat-clearing, diuresis promotion, detoxification, analgesia and urination facilitation [2,3,4]. *P. capitata* has been artificially cultivated for approximately 20 years, with the annual planting area reaching 1000 hectares in Guizhou. Agricultural cultivation practices have demonstrated that nitrogen fertilizer supplementation can effectively improve its biomass. However, excessive nitrogen input negatively affects the accumulation of bioactive ingredients, which may ultimately result in substandard medicinal quality. Such quality degradation has caused substantial economic losses to local cultivating farmers.

Flavonoids are the core chemical components of medicinal plants, involving a variety of biological activities. The biosynthesis of flavonoids is influenced by biotic and abiotic stresses, including light intensity, drought stress, bacteria and fungi [5,6]. In a study focusing on buckwheat, Wang et al. demonstrated that varying light intensities could accelerate the expression of flavonoids in addition to their key synthase genes [7]. In a similar study, Qin et al. reported that optimal light conditions facilitate plant growth and flavonoid accumulation in celery [8]. In an investigation into lignin and flavonoid metabolism in *Capsicum annuum*, Lv et al. reported that drought stress upregulates the expression levels of *PAL*, *C4H*, and *CHI* genes, thereby enhancing flavonoid biosynthesis [9]. From the perspective of biological stress, mycorrhizal fungus inoculation was also found to dramatically elevate flavonoid accumulation in *Anoectochilus roxburghii* [10].

Nitrogen fertilization has a marked influence on flavonoid biosynthesis in medicinal plants [11]. Research has demonstrated that nitrogen supplementation can effectively increase apple yield; in comparison, excessive nitrogen input lowers the carbon nitrogen ratio and thereby inhibits the accumulation of soluble sugars and total flavonoids in apple fruits [12]. Similar inhibitory effects of high nitrogen have been observed across multiple plant species: Li et al. [13] reported that low-to-moderate nitrogen levels are optimal for flavonoid accumulation in the leaves of *Coreopsis tinctoria*, and high nitrogen supply decreases flavonoid yields in *Centella asiatica* [14] and *Epimedium pilosum* [15]. Flavonoids are the main chemical components of *P. capitata* [16,17], and the results of previous studies have demonstrated that nitrogen application can reduce the content of flavonoids. However, the regulation mechanism of flavonoid biosynthesis in *P. capitata* under nitrogen application has yet to be elucidated. Understanding the regulation of nitrogen fertilizer on the growth and flavonoid metabolism of *P. capitata* is the key to optimizing its planting practice and improving its quality.

A limited number of research groups have explored how nitrogen regulates flavonoid biosynthesis in *P. capitata*. The present study was designed to investigate the effects of nitrogen fertilization on plant growth and flavonoid accumulation in *P. capitata*, in addition to clarifying the underlying regulatory mechanism of nitrogen-modulated flavonoid biosynthesis. Four nitrogen concentrations were established for comparative cultivation. We determined key indicators concerning the growth performance, biological traits, and yield of *P. capitata* across different nitrogen treatments. An integrated transcriptomic and metabolomic analysis was performed to screen pivotal functional genes and differential metabolites responsible for flavonoid synthesis. In this study, we aim to unravel the core regulatory network of flavonoid biosynthesis under nitrogen regulation, thereby providing a theoretical basis and reference for optimizing fertilization management and improving the medicinal quality of *P. capitata*.

## 2. Materials and Methods

### 2.1. Experimental Material

The plant materials consisted of one-year-old *P. capitata* seedlings propagated from seed, provided by Guizhou Warmen Pharmaceutical Co., Ltd., Guiyang, China. The test soil was yellow loam, with basic physicochemical properties as follows: pH 5.69, available nitrogen 48 mg/kg, available phosphorus 1.8 mg/kg, available potassium 74 mg/kg, and organic matter 33.9 g/kg. The test fertilizers were urea (N 46%, Guizhou Chitianhua Co., Ltd., Guiyang, China), calcium superphosphate (P_2_O_5_ 12%, Guizhou Qiantianhua Ecological Fertilizer Co., Ltd., Qiandongnan, China), and potassium sulfate (K_2_O 52%, Xinjiang Lop Nur Potash Co., Ltd., Bayingolin Mongolian Autonomous Prefecture, Xinjiang Uygur Autonomous Region, China). The experimental basin was 14 cm in height, with an upper edge diameter of 23.5 cm.

### 2.2. Experimental Design

The experiment was conducted from May to October 2025 in the greenhouse of Guizhou University of Traditional Chinese Medicine (106°61′ E, 26°39′ N) in Guiyang, China. A controlled pot experiment was performed, each pot was filled with 3 kg of dry loam soil sieved through a 2 mm mesh, and one seedling was planted per pot. Four nitrogen treatments were applied, with 10 pots per treatment: 0, 0.10, 0.20 and 0.30 N g/kg dry soil. The application of phosphorus and potassium fertilizers was consistent for each treatment, with a dosage of 0.15 g/kg dry soil. Before transplantation, the fertilizer was fully mixed with soil and the mixture was placed into pots. All other cultivation and management measures remained unchanged throughout the experiment.

### 2.3. Determination of Yield and Morphological Indicators

During the harvest period, plant morphological indicators were measured, including stem length and number of branches. Stem length was quantified with a ruler, followed by observation and documentation of branch quantities. The fresh weight of the plant was measured with an electronic balance (readability: 0.1 g; manufacturer: Shanghai Yingheng Electronic Scale Co., Ltd., Shanghai, China). After harvest, the plant material was dried and ground, and the leaf nitrogen content was determined using the Kjeldahl method [18].

### 2.4. Transcriptome Materials and Methods

In this study, four nitrogen application levels (CK, T1, T2, T3) were set up to investigate agronomic, physiological and phenotypic traits. Among them, T2 represented a moderate nitrogen supply with intermediate growth indexes, and its transcriptomic and metabolic profiles were expected to show a transitional state between low-nitrogen T1 and high-nitrogen T3. To reduce sequencing cost and redundant data, we selected the critical contrast groups including control (CK), low nitrogen (T1) and excessive high nitrogen (T3) for combined transcriptome and metabolome analysis, which covered the core gradient of nitrogen deficiency vs. nitrogen excess. In transcriptome analysis, leaf samples (0.5 g) of the CK, T1 and T3 groups collected at the harvest stage were sent to Metware Biotechnology Co., Ltd. (Wuhan, China) for RNA extraction and sequencing; four biological replicates were established for each treatment. The CTAB reagent was used to extract total RNA. Subsequently, a Qubit fluorescence quantitative analyzer (Invitrogen, Thermo Fisher Scientific, Waltham, MA, USA) was used to accurately quantify RNA, and the RNA integrity index (RQN value) was detected using a Qsep400 high-throughput biological fragment analyzer (BiOptic Inc., New Taipei City, Taiwan, China) to ensure that the sample quality met the library standards. mRNA was enriched with Oligo (dT) magnetic beads and a cDNA library was constructed. PE150 sequencing was performed on the Illumina NovaSeq platform, and the raw data was subjected to quality control and filtered obtain clean data. Trinity was used to assembling and splice transcripts. All raw RNA-seq reads were deposited in the NCBI Sequence Read Archive (SRA) under BioProject PRJN1513155 (https://www.ncbi.nlm.nih.gov/sra/PRJNA1513155) (accessed on 15 August 2026). Differentially expressed genes (DEGs) were analyzed using DESeq2 (version 1.22.2); a threshold of |log2 FC| ≥ 1 and an FDR < 0.05 were applied to identify DEGs. GO and KEGG enrichment analyses were performed on each comparison to determine the functions of DEGs.

### 2.5. Metabolome Materials and Methods

Leaf samples (0.5 g) of the CK, T1 and T3 groups collected at the harvest stage were used for metabolomic analysis. To ensure experimental accuracy, four biological replicates were established for each treatment. The samples were sent to Metware Biotechnology Co., Ltd. (Wuhan, China) for advanced extraction and analysis. The experimental sample was vacuum freeze-dried (Scientz-100F, Ningbo Scientz Biotechnology Co., Ltd., Ningbo, China) and then ground into powder using a grinder. Thereafter, 30 mg of the sample was weighted and added it to 1500 μL of pre-cooled (−20 °C) 70% methanol–water extraction solution. The mixture was vortexed every 30 min for a total of six cycles. After centrifugation, the supernatant was collected; the sample was filtered through a 0.22 μm microporous membrane, and then stored it in an injection vial for LC-MS/MS analysis.

All samples were acquired by the LC-MS system. The analytical conditions were as follows: UPLC: column, Waters ACQUITY UPLC HSS T3 1.8 µm, 2.1 mm × 100 mm; column temperature, 40 °C; flow rate, 0.40 mL/min; injection volume, 4 μL; solvent system, water (0.1% formic acid), acetonitrile (0.1% formic acid). Sample measurements were performed with a gradient program that employed the starting conditions of 95% A, 5% B. Within 5 min, a linear gradient to 35% A, 65% B was programmed. Within 1 min, a linear gradient to 1% A, 99% B was programmed, maintained for 1.5 min. Subsequently, a composition of 95% A, 5.0% B was adjusted within 0.1 min and maintained for 2.4 min. MS Conditions (AB): data acquisition was performed with the information-dependent acquisition (IDA) mode using Analyst TF 1.7.1 Software (Sciex, Concord, ON, Canada). The source parameters were set as follows: ion source gas 1 (GAS1), 50 psi; ion source gas 2 (GAS2), 60 psi; curtain gas (CUR), 35 psi; temperature (TEM), 550 °C, or 550 °C; declustering potential (DP), 80 V, or −80 V in positive or negative modes, respectively; and ion spray voltagefloating (ISVF), 5500 V or −4500 V in positive or negative modes, respectively.

Raw mass spectrometry data was converted to mzML format using ProteoWizard (version 3.0.21354-9ee14c7). Peak detection, alignment, and retention time correction were subsequently performed using the XCMS program. Peaks with a missing rate exceeding 50% within each treatment group were removed. Missing value imputation was conducted using a combined strategy: features with missing rates below 50% were imputed via the K-nearest neighbors (KNN) algorithm, whereas those with missing rates above 50% were filled with 1/5 of the minimum detected value. Peak area normalization was performed using the support vector regression (SVR) method. Metabolite identification was performed by searching the calibrated and filtered peaks against the self-built Metware metabolite database, integrated with public databases, prediction databases, and the MetDNA approach. Metabolites with a comprehensive identification score ≥ 0.5 and a QC sample CV value < 0.5 were retained. Positive and negative ionization modes were subsequently merged, preserving the metabolite entry with the highest qualitative confidence level and the smallest CV value. The data matrix was analyzed using R software (version 4.1.2). Univariate statistical analyses and multivariate data analyses were conducted. Model robustness was evaluated via seven-fold cross-validation and permutation tests. Metabolites with VIP ≥ 1 and FC ≥ 2 or fold change (FC) ≤ 0.5, together with *p*-value < 0.05, were considered statistically significant. Hierarchical clustering heatmap analyses were performed using the R package. Function annotation and enrichment analysis of DEMs was conducted using the KEGG database (http://www.kegg.jp/kegg/compound/ and https://www.kegg.jp/kegg/pathway.html) (accessed on 15 August 2026).

### 2.6. Statistic Analysis

Experimental data were processed and archived in Microsoft Excel. One-way analysis of variance (one-way ANOVA) was performed via IBM SPSS Statistics 21.0 to evaluate the effects of different nitrogen application rates on morphological traits and yield indices of *P. capitata*. Normality and homogeneity of variance were checked before statistic analysis. Duncan’s test was applied for multiple comparisons under homogeneous variance; otherwise, Welch’s ANOVA with Games–Howell pairwise comparisons was employed. Statistical significance was defined as *p* < 0.05. Figures were generated using Origin and the Metware Cloud Platform.

## 3. Results

### 3.1. The Effects of Different Nitrogen Fertilizers on the Growth Traits and Yield of P. capitata

**Figure 1:**
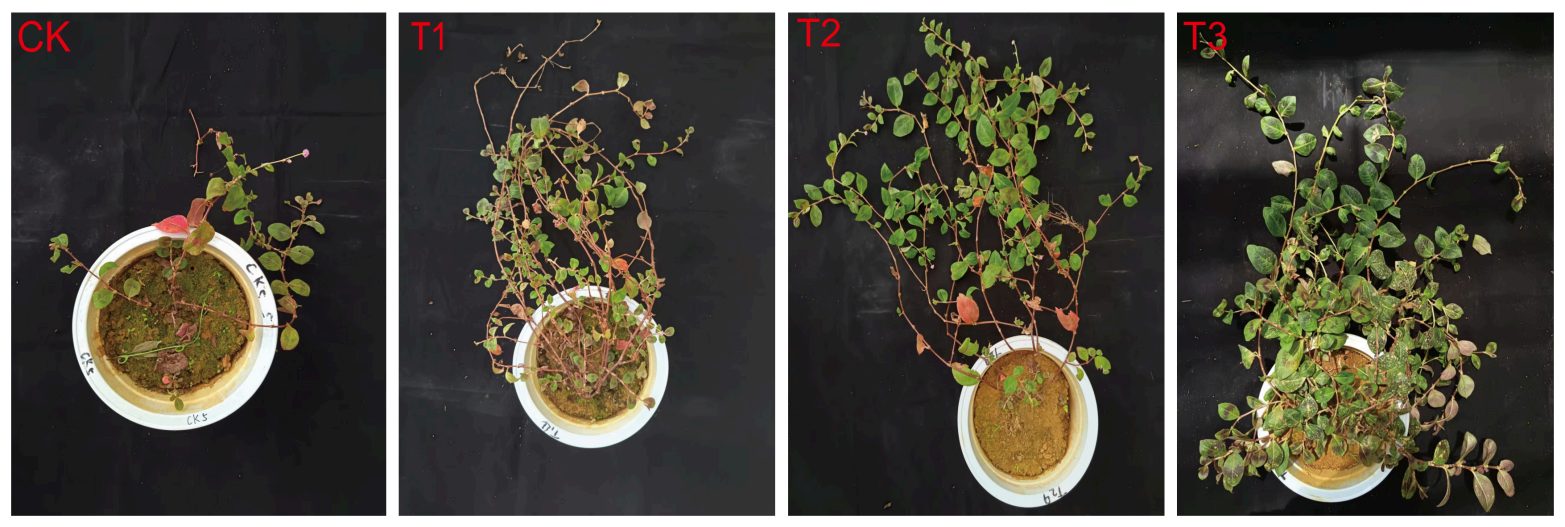
Phenotypic traits of *P. capitata* under different nitrogen treatments.

The phenotype analysis results indicate that nitrogen application markedly affected the growth of *P. capitata* (Figure 1). Overall, the biomass of *P. capitata* increased gradually with the elevation of nitrogen application levels. Plant morphological traits (stem length and branch number) and leaf nitrogen content differed significantly across treatments (Table 1), and all of these indicators presented a continuous upward trend as the nitrogen dosage increased. In terms of stem length, the T3 group yielded the maximum value of 80.74 cm, whereas no statistical difference was detected among the T1, T2, and T3 groups. The control group (CK) exhibited the shortest stem length (48.58 cm). In terms of branch number, the T3 treatment resulted in the highest value of 37.30, while no significant difference was observed between the T2 and T3 groups, and the CK group exhibited the lowest branch number (19.20). Additionally, the leaf nitrogen content peaked in the T3 group at 0.87%, followed by 0.84% in the T2 group, with an insignificant difference between the two treatments; the CK group possessed the lowest leaf nitrogen content of 0.52%. In terms of yield, the T3 group achieved the highest value of 123.64 g/plant. No significant difference was found between the T1 and T2 groups, and the CK group exhibited the lowest yield of 14.2 g/plant.

### 3.2. Transcriptome Profiling Under Different Nitrogen Application Rates

Transcriptome sequencing was performed on three groups of samples. A total of 103.79 GB of clean data was obtained from 12 samples, with each sample exceeding 7.68 GB. The Q30 bases ratio of each sample exceeded 97%, and the GC content ranged from 46.44% to 46.95% (Table S1), indicating high sequencing accuracy. Trinity was used to concatenate clean reads. A total of 127,986 transcripts were assembled, with an average length of 1672 bp, N50 length of 2597 bp, unigene length of 80,072, average length of 2001 bp, and N50 length of 2844 bp (Table S2). BUSCO assessment yielded a high assembly completeness of 96.50%, which validated the excellent integrity and reliability of the de novo transcriptome assembly for the CK, T1, and T3 groups (Figure S1). Subsequent read alignment analysis demonstrated that the total mapping rate of clean reads matched to the assembled unigenes was higher than 92.88% (Table S3). Gene function annotation was performed using Unigene, KEGG, GO, Nr, SwissProt, and KOG, Which were used to annotate 49.62% (39,732), 66.40% (53,168), 74.49% (59,642), 59.37% (47,541), and 48.16% (38,563), respectively (Table S4).

#### 3.2.1. Differentially Expressed Gene Analysis

**Figure 2:**
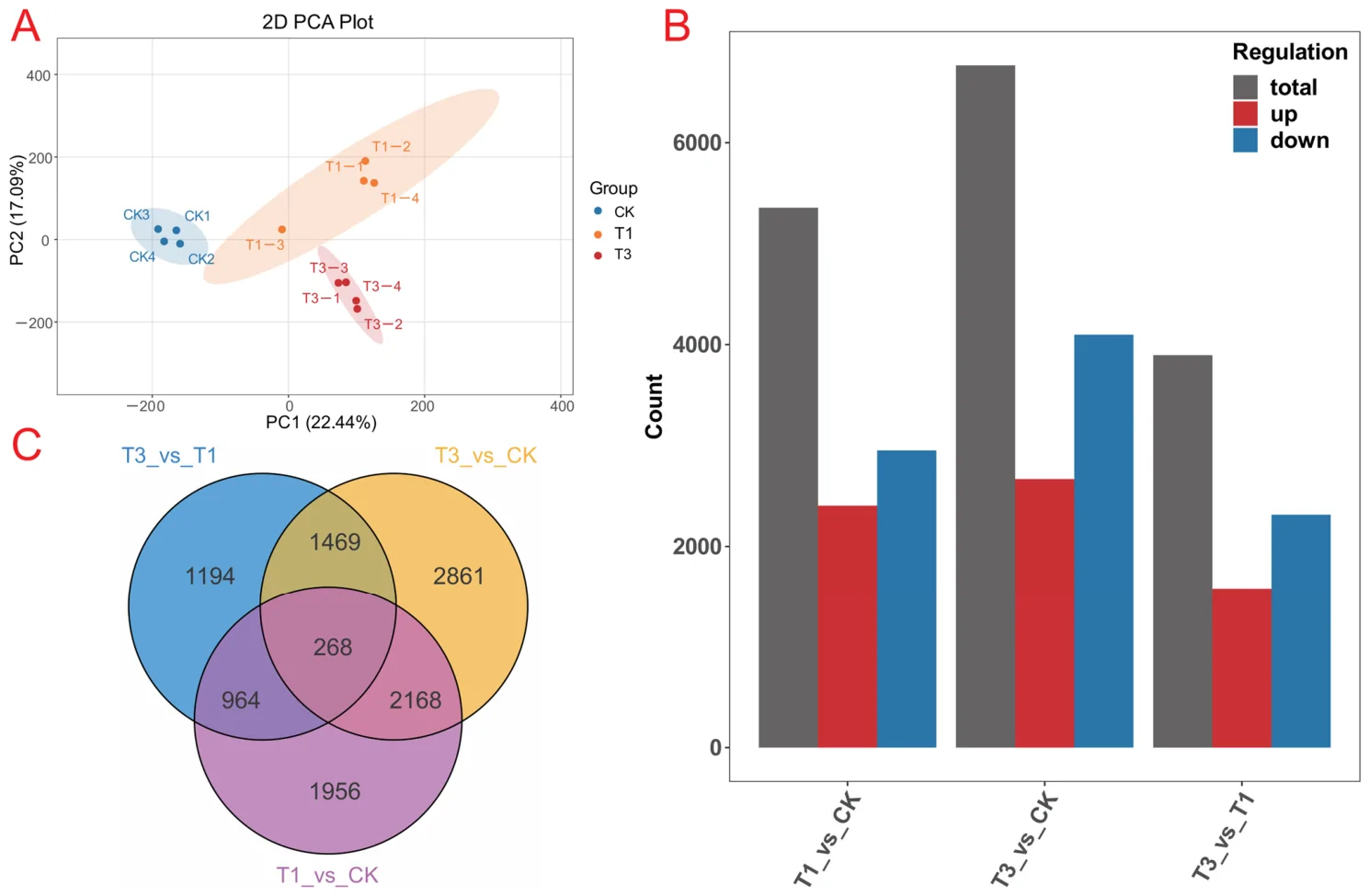
PCA plot (**A**), bar chart (**B**), and Venn diagram (**C**) of differentially expressed genes across different treatments.

The results of principal component analysis showed that the differences between the two principal components were significant, accounting for 22.44% and 17.09% of the total variance, respectively. Biological replicates clustered tightly together, whereas samples from different nitrogen treatments were clearly separated, indicating that nitrogen application markedly altered the leaf transcriptomic characteristics (Figure 2A). Differential expression analysis was conducted based on the threshold values of |log2 fold change| ≥ 2 and *p* < 0.05. A total of 10,880 differentially expressed genes (DEGs) were identified across the three comparison groups. Specifically, 5356 DEGs were screened in the T1_vs_CK comparison, including 2404 upregulated and 2952 downregulated genes. The T3_vs_CK comparison yielded 6766 DEGs, consisting of 2688 upregulated and 4098 downregulated genes. In addition, 3832 DEGs were detected in the T3_vs_T1 group, with 1508 upregulated and 2315 downregulated genes (Figure 2B). Further analysis revealed that the number of DEGs increased with the elevation of nitrogen application, and downregulated DEGs were consistently more abundant than their upregulated counterparts across all comparison groups. A Venn diagram analysis was then performed to identify shared and unique DEGs across all groups. A total of 268 common DEGs were found to be simultaneously expressed in all three comparison groups (Figure 2C). In addition, 1956, 2861, and 1194 unique DEGs were specifically detected in the T1_vs_CK, T3_vs_CK, and T3_vs_T1 groups, respectively, implying that distinct transcriptional regulatory programs were activated under different nitrogen supply levels.

#### 3.2.2. GO and KEGG Enrichment Analysis of Differentially Expressed Genes

**Figure 3:**
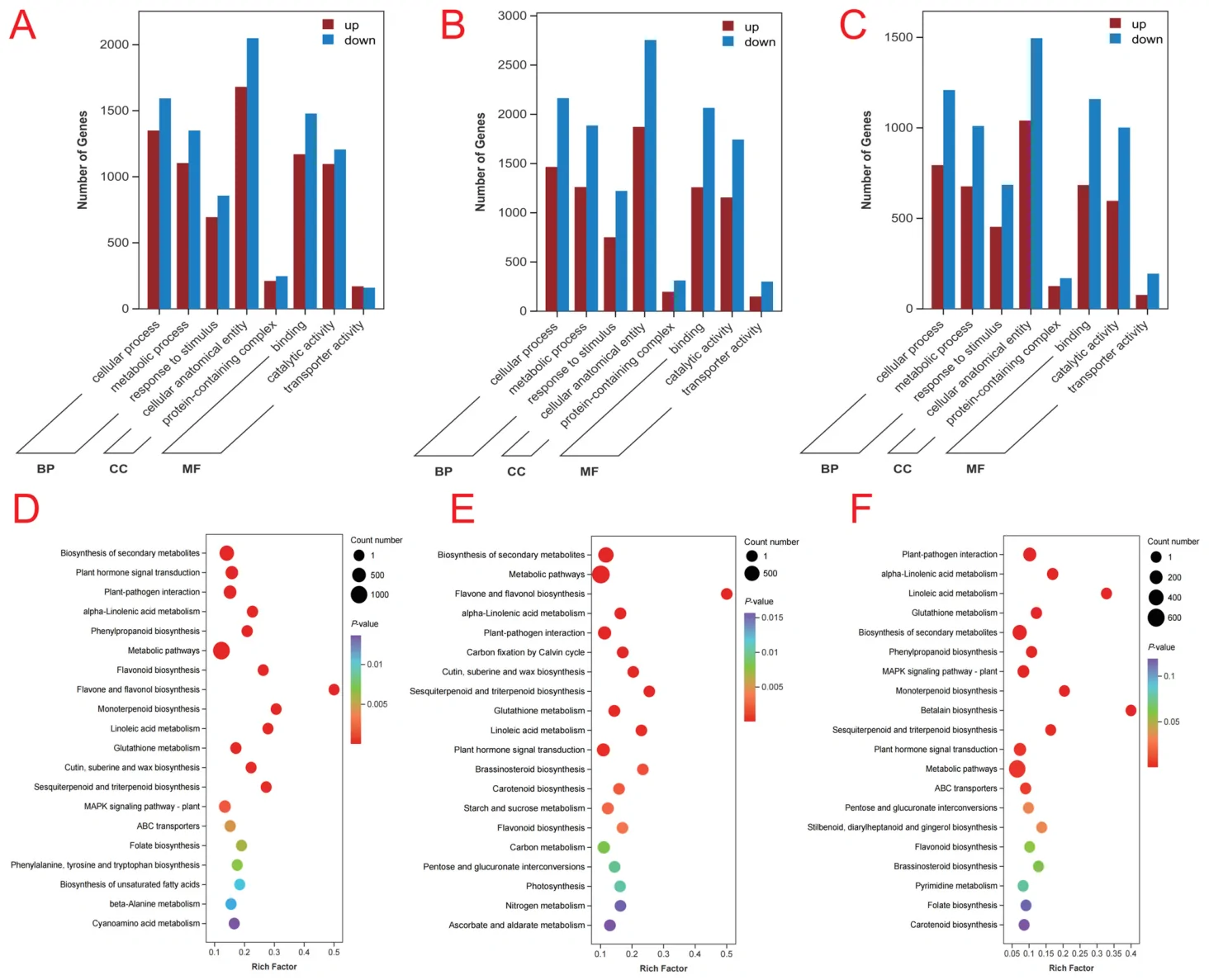
GO and KEGG enrichment analysis plots ((**A**–**C**) represent the GO enrichment analysis; (**D**–**F**) represent the KEGG enrichment analysis).

The top enriched GO terms for biological process (BP), cellular component (CC), and molecular function (MF) across the three comparison groups are displayed in Figure 3A–C. Notably, all groups exhibited highly consistent GO enrichment profiles. In the BP category, cellular process and metabolic process were the most significantly enriched terms, followed by response to stimulus and biological regulation. In terms of the CC category, cellular anatomical entity and protein-containing complex were predominantly enriched. In terms of MF, binding and catalytic activity represented the two most highly enriched functional terms. KEGG pathway enrichment analysis was further conducted to explore the key metabolic pathways involved in nitrogen response. The top 20 significantly enriched pathways are summarized in Figure 3D–F. The core enriched pathways included metabolic pathways, biosynthesis of secondary metabolites, and plant hormone signal transduction. Importantly, several flavonoid metabolism-related pathways closely associated with our study were significantly enriched, including phenylpropanoid biosynthesis (ko00940), flavonoid biosynthesis (ko00941), and flavone and flavonol biosynthesis (ko00944).

#### 3.2.3. Differentially Expressed Genes in Flavonoid Biosynthesis

DEGs associated with flavonoid biosynthesis in *P. capitata* were further characterized, with the enrichment results presented in Table 2. Among the annotated metabolic pathways, phenylpropanoid biosynthesis exhibited the highest enrichment of flavonoid-related DEGs, followed by flavonoid biosynthesis and flavone and flavonol biosynthesis pathways. In the Phenylpropanoid biosynthesis metabolism pathway, 43 DEGs (20 upregulated and 23 downregulated), 74 DEGs (17 upregulated and 57 downregulated), and 38 DEGs (9 upregulated and 29 downregulated) were enriched in the T1_vs_CK, T3_vs_CK, and T3_vs_T1 comparisons, respectively. In the flavonoid synthesis metabolism pathway, 20 DEGs (7 upregulated and 13 downregulated), 31 DEGs (6 upregulated and 25 downregulated), and 12 DEGs (2 upregulated and 10 downregulated) were enriched in the three comparison groups in sequence. Additionally, the flavone and flavonol biosynthesis pathway included 10 DEGs (7 upregulated and 3 downregulated) in T1_vs_CK, 10 DEGs (6 upregulated and 4 downregulated) in T3_vs_CK, and only one upregulated DEG in T3_vs_T1.

### 3.3. Untargeted Metabolic Profiling of Different Treatments

#### 3.3.1. Metabolite Accumulation Analysis Under Different Nitrogen Application Rates

**Figure 4:**
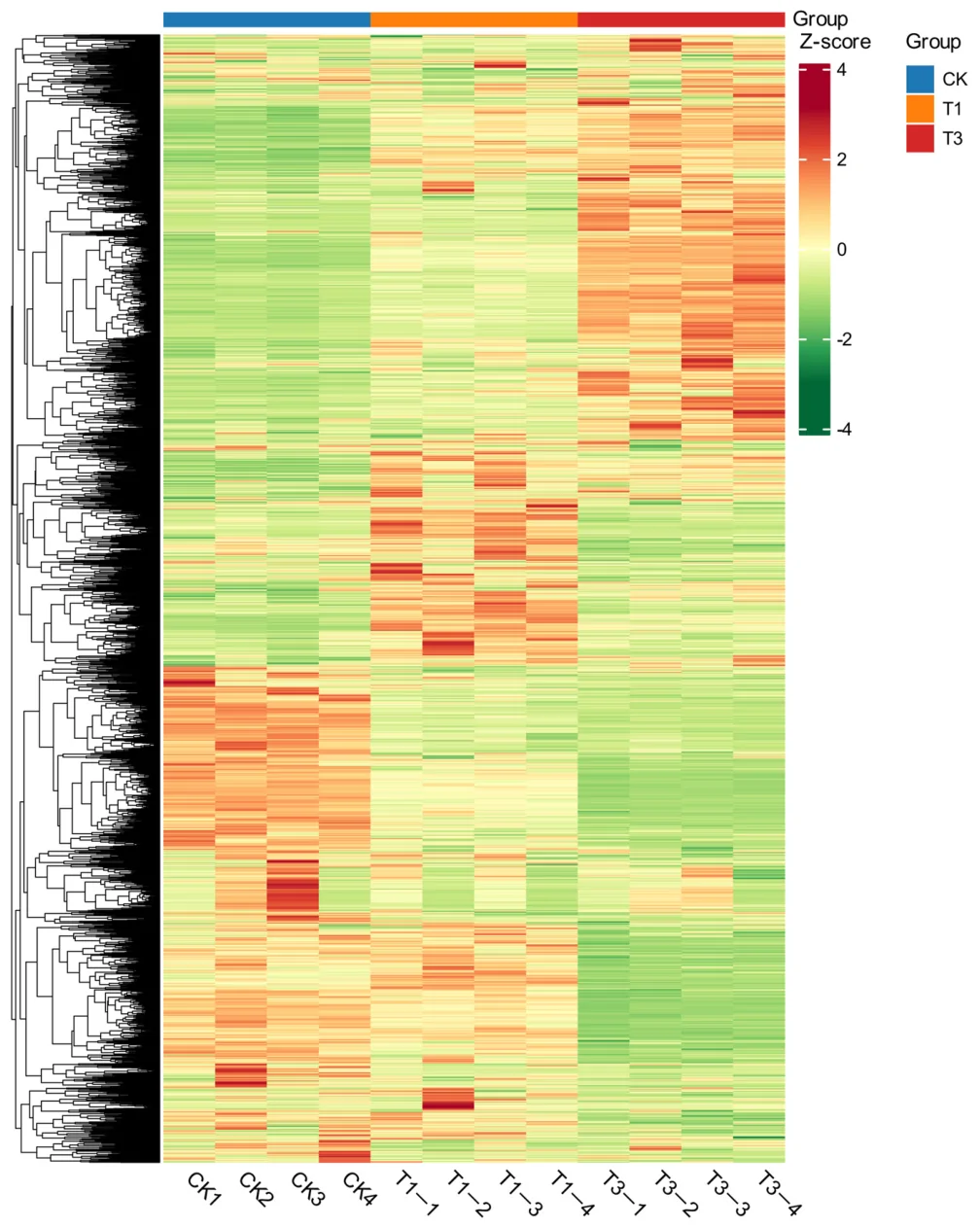
Metabolite heatmaps of different treatments.

To elucidate the effects of nitrogen treatment on metabolite accumulation in *P. capitata* leaves, untargeted metabolomics analysis was conducted on three sample groups. A total of 3356 metabolites were identified in *P. capitata* leaves, The predominant metabolites consisted of organic acids (24.73%), amino acids and their derivatives (14.67%), benzene ring derivatives (10.37%), flavonoids (7.78%), as well as phenolic acids (3.48%) (Figure S2). In addition, other annotated metabolites mainly included terpenoids, tannins, and nucleotides and their derivatives. Principal component analysis (PCA) revealed distinct metabolic profiles among samples subjected to different nitrogen levels. PC1 and PC2 explained 42.21% and 17.75% of the total variance, respectively, accounting for a cumulative contribution of 59.96% (Figure S3). Moreover, the hierarchical clustering heatmap demonstrated obvious inter-group discrepancies and favorable biological repeatability within each treatment (Figure 4).

#### 3.3.2. Differentially Metabolites and KEGG Enrichment Analysis

Differentially accumulated metabolite (DAM) analysis across different treatment groups revealed a total of 924 DAMs in the T1_vs_CK comparison, among which 647 were significantly upregulated and 277 were significantly downregulated. The T3_vs_CK comparison identified 1528 DAMs, consisting of 748 upregulated and 780 downregulated metabolites. Additionally, a total of 1096 DAMs were screened in the T3_vs_T1 comparison, including 361 significantly upregulated and 735 significantly downregulated metabolites (Table 3). As the nitrogen application rate increased, the quantity of downregulated DAMs rose progressively.

**Figure 5:**
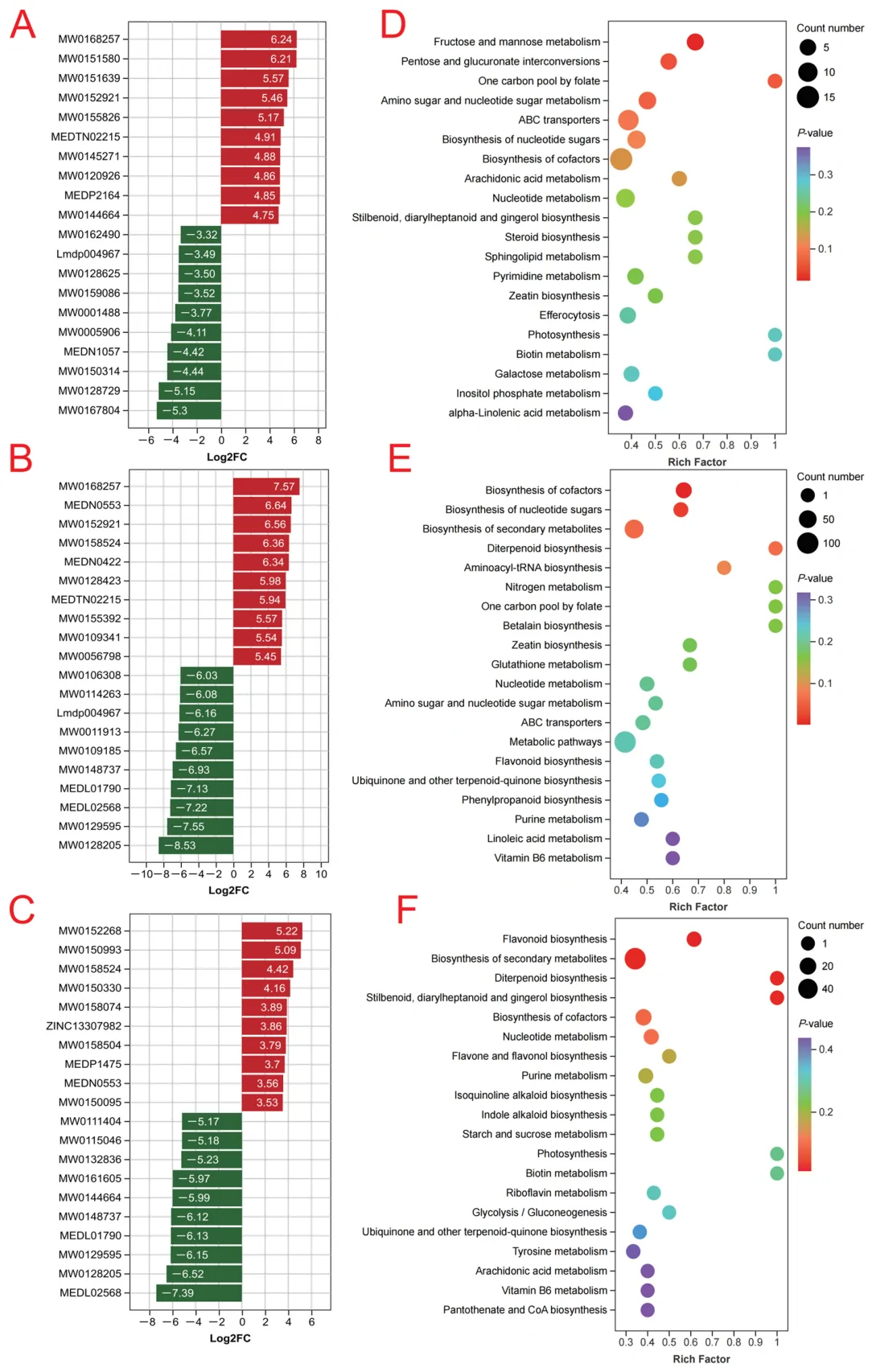
Top 20 differentially accumulated metabolites ((**A**): T1_vs_CK; (**B**): T3_vs_CK; (**C**): T3_vs_T1. The correspondence between indices and substances is shown in Tables S5–S7) and KEGG enrichment pathways under different nitrogen treatments ((**D**): T1_vs_CK; (**E**): T3_vs_CK; (**F**): T3_vs_T1).

The top 20 upregulated and downregulated DAMs ranked by fold change (Log2FC) values in each comparison group are visualized in Figure 5A–C. In the T1_vs_CK group, 2,3-dimercaptosuccinic acid (Log2FC = 6.24) exhibited the greatest upregulation, whereas (Z)-octadec-9-enoate (Log2FC = −5.30) showed the most significant downregulation. For the T3_vs_CK comparison, 2,3-dimercaptosuccinic acid (Log2FC = 7.57) exhibited the greatest upregulation, while oxidanesulfonic acid (Log2FC = −8.53) presented the most significant downregulation. In the T3_vs_T1 group, Leu-Asp-Pro-Asp-Tyr (Log2FC = 5.22) was the most significantly upregulated metabolite, and FAA (17:1) (Log2FC = −7.39) was the most markedly downregulated metabolite. Further functional classification demonstrated that the significantly upregulated metabolites were primarily composed of amino acids and their derivatives, whereas the downregulated metabolites mainly encompassed flavonoids, organic acids, and phenolic acids.

The KEGG results demonstrated that the fructose and mannose metabolism pathway was significantly enriched in the T1_vs_CK group, whereas the biosynthesis of cofactors and biosynthesis of nucleotide sugars pathways were significantly enriched in the T3_vs_CK group. For the T3_vs_T1 comparison, the markedly enriched pathways included flavonoid biosynthesis, biosynthesis of secondary metabolites, diterpenoid biosynthesis, and stilbenoid, diarylheptanoid and gingerol biosynthesis (Figure 5D–F). Notably, the flavonoid biosynthesis-related pathways covered three core metabolic pathways, namely, phenylpropanoid biosynthesis (ko00940), flavonoid biosynthesis (ko00941), and flavonoid and flavonol biosynthesis (ko00944).

#### 3.3.3. Differentially Accumulated Flavonoids Analysis

**Figure 6:**
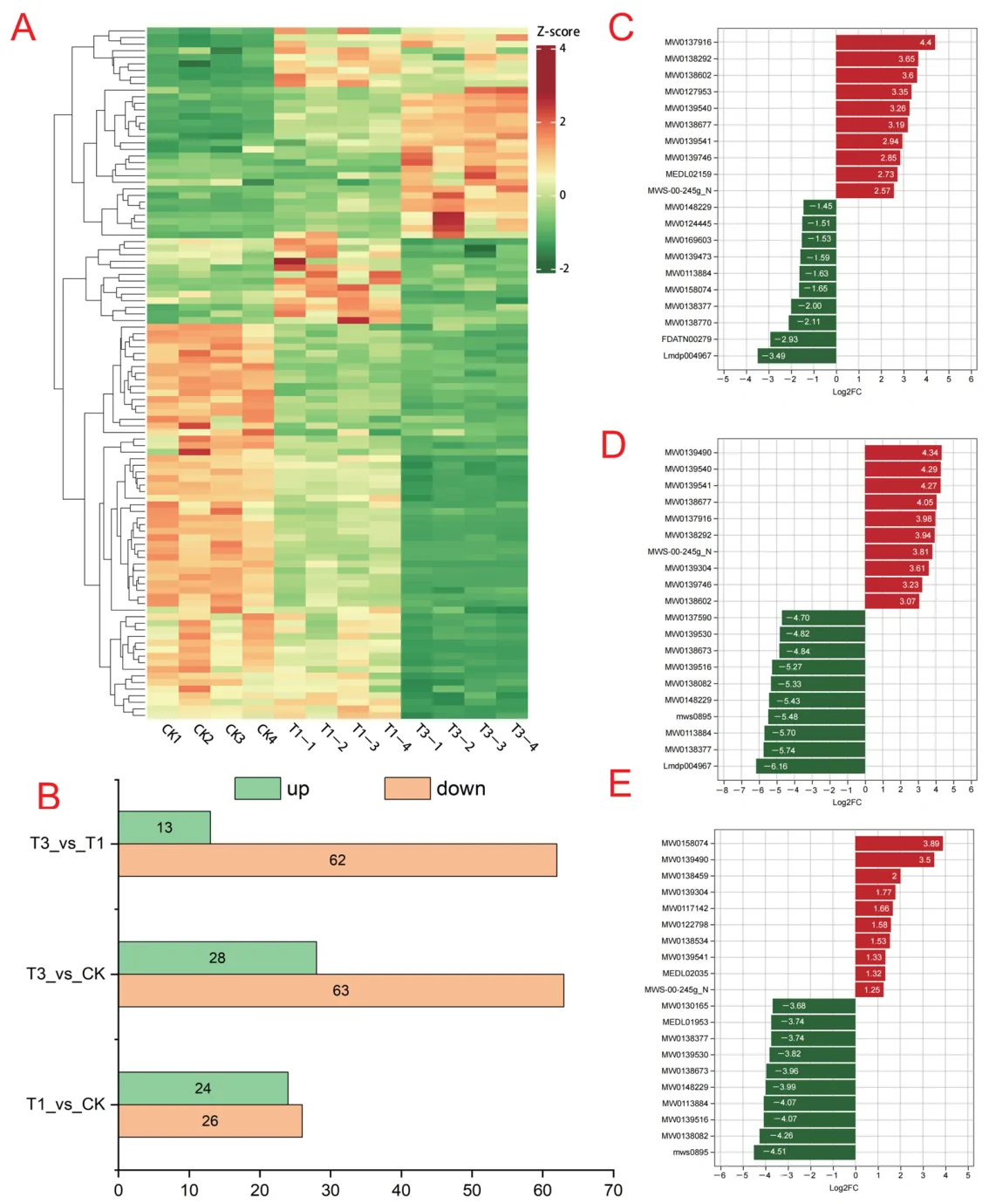
(**A**): Heatmap of differential flavonoid-accumulated metabolites; (**B**): up- and downregulated flavonoid metabolites in different treatment groups; (**C**–**E**): top 20 differential flavonoid metabolites in T1_vs_CK, T3_vs_CK, and T3_vs_T1 treatments (the correspondence between indices and substances is shown in Tables S8–S10).

Flavonoids are the main active ingredients of *P. capitata*. In the present study, a total of 3356 metabolites were identified, among which 168 were identified as flavonoids and their derivatives. These flavonoids comprised 60 flavonoids, 31 flavonols, and 18 other flavonoids, accounting for 35.71%, 18.45%, and 10.71% of the total flavonoid metabolites, respectively (Table 4). The hierarchical clustering heatmap indicates that the expression profiles of each group of samples are clearly separated, with high consistency within the same group (Figure 6A). Additionally, most flavonoid metabolites exhibited an overall downregulated expression trend in the treatment groups. Comparing the flavonoids in different treatments, it was found that there were 105 significantly differentially accumulated flavonoids (DAFs) among the three treatments. Specifically, in T1_vs_CK, a total of 50 DAFs were detected, including 24 upregulated and 26 downregulated metabolites. For T3_vs_CK, 91 DAFs were detected, consisting of 28 upregulated and 63 downregulated compounds. Moreover, the T3_vs_T1 comparison yielded 75 DAFs, with only 13 metabolites upregulated and 62 metabolites significantly downregulated (Figure 6B).

The bar chart illustrates the top 20 significantly up- and downregulated DAFs in the three comparative groups based on fold change (Figure 6C–E). In T1_vs_CK, Delphinidin-3-o-arabinoside exhibited the greatest upregulation (log2FC = 4.40), whereas Kaempferol-3-O-glucoside showed the most significant downregulation (log2FC = −3.49). For the T3_vs_CK comparison, 2-(3,4-dihydroxyphenyl) presented the greatest upregulation (log2FC = 4.40), and kaempferol-3-O-glucoside showed the greatest downregulation (log2FC = −6.16). In T3_vs_T1, Tripteroside exhibited the greatest upregulation (log2FC = 3.89), whereas Genistin showed the most significant downregulation (log2FC = −4.51). Notably, quercitrin, an indicator component of *P. capitata*, was also significantly downregulated (log2FC = −1.59).

### 3.4. Integrated Analysis of Transcriptomics and Metabolomics

**Figure 7:**
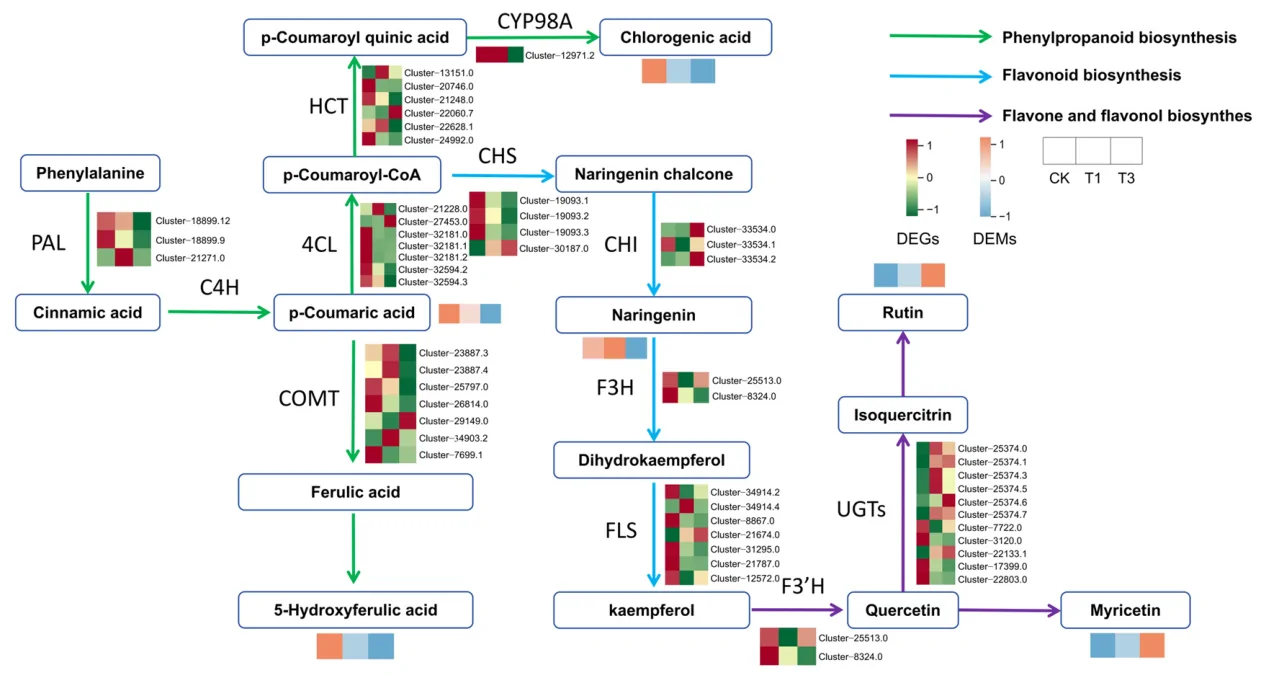
Metabolic pathway of flavonoids in *P. capitata* under nitrogen application.

Integrated analysis of transcriptomics and metabolomics was performed to systematically explore the relationships between DEGs and DAMs from the different nitrogen treatments. Among the enriched metabolic pathways, those involved in flavonoid biosynthesis included the phenylpropanoid pathway (ko00940), flavonoid biosynthesis (ko00941) and flavone and flavonol metabolism pathway (ko00944). To clarify the variations in genes and metabolites in the flavonoid biosynthesis pathway of *P. capitata* leaves after nitrogen treatment, a pathway map was created (Figure 7). The identified enzymes include *PAL* (phenylalanine ammonia-lyase, K10775), *4CL* (4-coumarate-CoA ligase, K01904), *HCT* (shikimate O-hydroxycinnamoyltransferase, K13065), *COMT* (caffeic acid 3-O-methyltransferase, K13066), *CYP98A* (5-O-(4-coumaroyl)-D-quinate 3′-monooxygenase, K09754), *CHS* (chalcone synthase, K00660), *CHI* (chalcone isomerase, K01859), *F3H* (naringenin 3-dioxygenase, K00475), *FLS* (flavonol synthase, K05278), *F3′H* (flavonoid 3′-monooxygenase, K05280), and *UGTs* (flavonol 3-O-glucosyltransferase, K10757). Additionally, differentially expressed metabolites include 4-hydroxycinnamic acid, chlorogenic acid, 5-hydroxyferulic acid, naringenin, cianidanol, epigallocatechin, taxifolin, luteolin, (+)-gallocatechin, and myricetin.

In the phenylpropanoid biosynthesis pathway, excluding cluster-21271.0, cluster-27453.0, and 21228.0, the expression levels of genes encoding *PAL* and *4CL* were downregulated with increasing nitrogen application. In the flavonoid synthesis pathway, most CHS-encoding genes exhibited decreased expression under elevated nitrogen conditions, with only Cluster-30178 being upregulated. In terms of *CHI* genes, one gene was downregulated and two were upregulated in response to increasing nitrogen application. The genes regulating *F3H* were all downregulated, while the genes regulating *FLS*, except cluster-21674.0, were downregulated with increasing nitrogen application. In the Flavone and flavonol biosynthesis pathway, all F3′H-encoding genes were downregulated under increasing nitrogen application. Additionally, among the *UGT* family genes, seven were downregulated and four were upregulated following increased nitrogen application.

Regarding differential metabolites, the accumulation of p-coumaric acid decreased gradually with increasing nitrogen application, which was consistent with the expression trend of *PAL* genes. Similarly, chlorogenic acid and 5-hydroxyferulic acid also displayed a nitrogen-dependent declining pattern, which was in line with the downregulated expression of key structural genes involved in phenylpropanoid biosynthesis, including *4CL*, *HCT*, and *COMT*. In the flavonoid metabolic pathway, multiple core metabolites, namely, naringenin, cianidanol, epigallocatechin, taxifolin, luteolin, and (+)-gallocatechin, exhibited reduced accumulation under elevated nitrogen conditions. In contrast, the contents of myricetin and rutin increased significantly with the rise in nitrogen application rate.

## 4. Discussion

### 4.1. The Effect of Nitrogen Fertilizer on the Growth of P. capitata

Nitrogen is an essential nutrient for plant growth and can substantially enhance the development of medicinal plants [19]. In the present study, nitrogen application markedly improved the biomass of *P. capitata* relative to the control (CK) treatment. The biomass of *P. capitata* increased gradually with the elevation of nitrogen dosage, and the yield under the T3 treatment was 8.7-fold higher than that of the CK group. Plant stem length and branch number exhibited a consistent variation trend with biomass yield. These findings are in line with previous research by Xing et al. [20], who reported that nitrogen fertilization significantly enhances the branch number, plant weight, maximum root length, and stem and root diameter of *Salvia miltiorrhiza*. Similar positive effects of nitrogen application on biomass accumulation have also been documented in *Valeriana amurensis* [21], *Ziziphora clinopodiides* [22] and *Pelargonium sidoides* [23].

### 4.2. Effects of Different Nitrogen Rates on the Biomass and Flavonoid Secondary Metabolites of P. capitata

As an herbaceous plant and one of the top ten Miao medicinal plants, *P. capitata* is widely planted in Guizhou. The results of this study indicate that nitrogen application significantly enhances the leaf nitrogen content and yield of *P. capitata*, an observation also made in other plants, such as *Liriope muscari* [24], olion [25] and *Tripterygium wilfordii* [26]. Metabolomics analysis demonstrated that the metabolites in the leaves of *P. capitata* are mainly include organic acids, amino acids and their derivatives, benzene rings, flavonoids, and phenolic acids. Flavonoids are the core pharmacological components of *P. capitata*, and a total of 168 differential metabolites of flavonoids were identified under different nitrogen treatments. With the increase in nitrogen application, flavonoids are mainly downregulated. Compared with CK, 24 flavonoids were upregulated and 26 flavonoids were downregulated in T1, and 28 flavonoids were upregulated and 63 flavonoids were downregulated in T3. This observation is consistent with established evidence that high nitrogen availability reduces the accumulation of flavonoids in plants [27]. With the increase in nitrogen application rate, the biomass of *P. capitata* gradually increases. Nitrogen-containing compounds such as alkaloids were significantly upregulated, while carbon-containing compounds such as flavonoids were significantly downregulated. This phenomenon can be attributed to the reduction in plant C/N ratio. According to the carbon nutrient balance hypothesis [28,29], flavonoid biosynthesis relies on L-phenylalanine derived from the shikimate pathway, an intermediate that also acts as a shared precursor for amino acid and protein production. Consequently, primary and secondary metabolism compete for photosynthetic carbon skeletons. Under low-nitrogen conditions, limited nitrogen availability elevates the plant C/N ratio and restricts vegetative growth, allowing carbohydrates (sucrose and starch) to accumulate in surplus. Excess carbon skeletons are then redirected toward carbon-based secondary metabolites of the phenylpropanoid pathway, including flavonoids, which ultimately elevate flavonoid concentrations. By contrast, sufficient nitrogen supply lowers the C/N ratio; plants prioritize allocating carbon resources to synthesize amino acids, proteins, and chlorophyll to sustain rapid vegetative growth. As photosynthate is largely consumed by primary metabolic processes, fewer carbon precursors are available for flavonoid biosynthesis, thereby suppressing flavonoid accumulation [30]. Consistent findings have been documented in *Epimedium* [15], in which rising nitrogen input reduced plant C/N ratios and subsequently downregulated flavonoid production.

### 4.3. Analysis of the Downregulation Mechanism of Flavonoids

In plants, the biosynthesis of flavonoids involves the phenylpropanoid, flavonoid, and flavone and flavonol metabolism pathways [31,32]. The results of this study indicate that compared with CK, T1 had 43 DEGs (20 upregulated, 23 downregulated) and T3 had 74 DEGs (17 upregulated, 57 downregulated) in phenylpropanoid metabolism. In the flavonoid metabolism pathway, compared with CK, T1 had 20 DEGs (7 upregulated, 13 downregulated) and T3 had 31 DEGs (6 upregulated, 25 downregulated). The DEGs in the phenylpropanoid and flavonoid metabolism pathway are mainly downregulated. In the flavone and flavonol metabolism pathway, compared with CK, both T1 and T3 have 10 DEGs, with 7 upregulated and 3 downregulated in T1 and 6 upregulated and 4 downregulated in T3. The differentially expressed genes are mainly upregulated. Combined analysis of transcription and metabolism showed that the key genes detected in the phenylpropanoid metabolism pathway include *PAL*, *4CL*, *HCT*, and *COMT*, among which *PAL* and *4CL* are the key rate-limiting enzymes for phenylpropane synthesis [22]. Compared with CK, high nitrogen application resulted in downregulation of coumaric acid, chlorogenic acid, and 5-hydroxyferulic acid in metabolites, potentially attributed to the downregulation of *PAL* and *4CL* genes, which aligns with the findings of Xu et al. [33]. who discovered that high-nitrogen conditions lead to the downregulation of “Fuji”apple structural genes, such as *MdPAL* and *Md4CL*, ultimately resulting in a decrease in anthocyanins and other flavonoids. In the flavonoid synthesis pathway, detected genes include *CHS*, *CHI*, *F3H*, and *FLS*. *CHS* is the rate-limiting enzyme in the biosynthesis of flavonoids [26]. The downregulation of *CHS*, *F3H*, and *FLS* genes may indirectly reduce the accumulation of multiple metabolites, including naringenin, catechin, epicatechin, quercetin, epigallocatechin gallate and luteolin. Furthermore, the decreased expression of *FLS* and *F3′H* contributes to the reduction in quercetin, a characteristic bioactive component in *P. capitata*. In addition, *UGTs* can catalyze the glycosylation of quercetin to form rutin, and the metabolic transformation of quercetin into myricetin is also responsible for the decline in quercetin content.

## 5. Conclusions

In the present study, we aimed to investigate the effects of nitrogen application on the growth, yield, and flavonoid biosynthesis of *P. capitata*. The results showed that nitrogen application significantly promoted growth and increased the yield, but significantly reduced the flavonoid content. Through a combined analysis of transcriptomics and metabolomics, it was found that the flavonoid metabolic pathways involved in *P. capitata* include the phenylpropanoid, flavonoid, and flavone and flavonol metabolic pathways. *PAL*, *4CL*, *CHS*, *FLS*, *F3H*, *F3′H*, and *UGTs* were involved in regulating the biosynthesis of flavonoids (such as chlorogenic acid, naringin, and quercetin). In this study, we elucidated the regulatory mechanism of flavonoid metabolites in *P. capitata* under nitrogen application, providing theoretical guidance for scientific fertilization of *P. capitata*. However, further research is required to investigate the biological functions of the genes regulating the flavonoid synthesis in *P. capitata*. Future work will focus on the effects of nitrogen application on the yield and quality of *P. capitata* under field conditions, selecting the optimal nitrogen application rate and providing a reference for the sustainable development of the *P. capitata* industry.

## Figures and Tables

**Table 1:** Differences in morphological traits and yield under different nitrogen treatments.

Treatment	Stem Length (cm)	Branch Number	Leaf Nitrogen Content (%)	Yield (g/Plant)
CK	48.58 ± 9.23 b	19.20 ± 8.38 c	0.52 ± 0.03 c	14.20 ± 7.73 c
T1	73.84 ± 15.90 a	30.60 ± 3.17 b	0.59 ± 0.02 b	66.37 ± 26.11 b
T2	80.54 ± 19.44 a	31.60 ± 4.74 ab	0.84 ± 0.05 a	70.04 ± 38.19 b
T3	80.74 ± 13.11 a	37.30 ± 9.07 a	0.87 ± 0.01 a	123.64 ± 32.32 a

Notes: Values with different lowercase letters within the same column are significantly different at *p* < 0.05.

**Table 2:** Differentially expressed genes in flavonoid metabolic pathways.

Metabolic Pathway	T1_vs_CK		T3_vs_CK		T3_vs_T1	
	Up	Down	Up	Down	Up	Down
Phenylpropanoid biosynthesis	20	23	17	57	9	29
Flavonoid biosynthesis	7	13	6	25	2	10
Flavone and flavonol biosynthesis	7	3	6	4	1	0

**Table 3:** Statistical analysis of differential metabolites between different treatments.

Group	All Significant Metabolites	Downregulated	Upregulated
T1_vs_CK	924	277	647
T3_vs_CK	1528	780	748
T3_vs_T1	1096	735	361

**Table 4:** Classification of flavonoid metabolites under different treatments.

Classification	Count	Portion
Flavones	60	35.71%
Flavonols	31	18.45%
Other Flavonoids	18	10.71%
Isoflavones	15	8.93%
Chalcones	13	7.74%
Flavanols	11	6.55%
Dihydroisoflavones	10	5.95%
Anthocyanidins	5	2.98%
Flavanonols	2	1.19%
Biflavones	2	1.19%
Dihydroisoflavones	1	0.60%

## Data Availability

The data presented in this study are available on request from the corresponding author. The data are not publicly available due to privacy restrictions.

## Supplementary Materials

The following supporting information can be downloaded at https://www.mdpi.com/article/10.3390/metabo16090616/s1. Figure S1. BUSCO completeness chart; Figure S2. Classification diagram of metabolites; Figure S3. PCA score plot of metabolites; Table S1. Sequencing results of different treatments; Table S2. Statistical table of assembly results; Table S3. Statistical table comparing clean reads of each sample to transcripts; Table S4. Annotations of differential expressed genes in different databases; Table S5. Top 20 differential metabolites between T1_vs_CK; Table S6. Top 20 differential metabolites between T3_vs_CK; Table S7. Top 20 differential metabolites between T3_vs_T1; Table S8. Top 20 flavonoid differential metabolites between T1_vs_CK; Table S9. Top 20 flavonoid differential metabolites between T3_vs_CK; Table S10. Top 20 flavonoid differential metabolites between T3_vs_T1.
