# Supplementary material for: Integrated Transcriptomics and Metabolomics to Reveal the Regulatory Mechanisms of Flavonoid Biosynthesis in Persicaria capitata Under Different Nitrogen Fertilization Levels
Source: Metabolites. 2026 Aug 27;16(9):616. doi: 10.3390/metabo16090616 (PMC13609273; doi:10.3390/metabo16090616)
Supplement: Supplementary file 1 [file metabolites-16-00616-s001.zip › metabolites-4488447-supplementary.pdf]

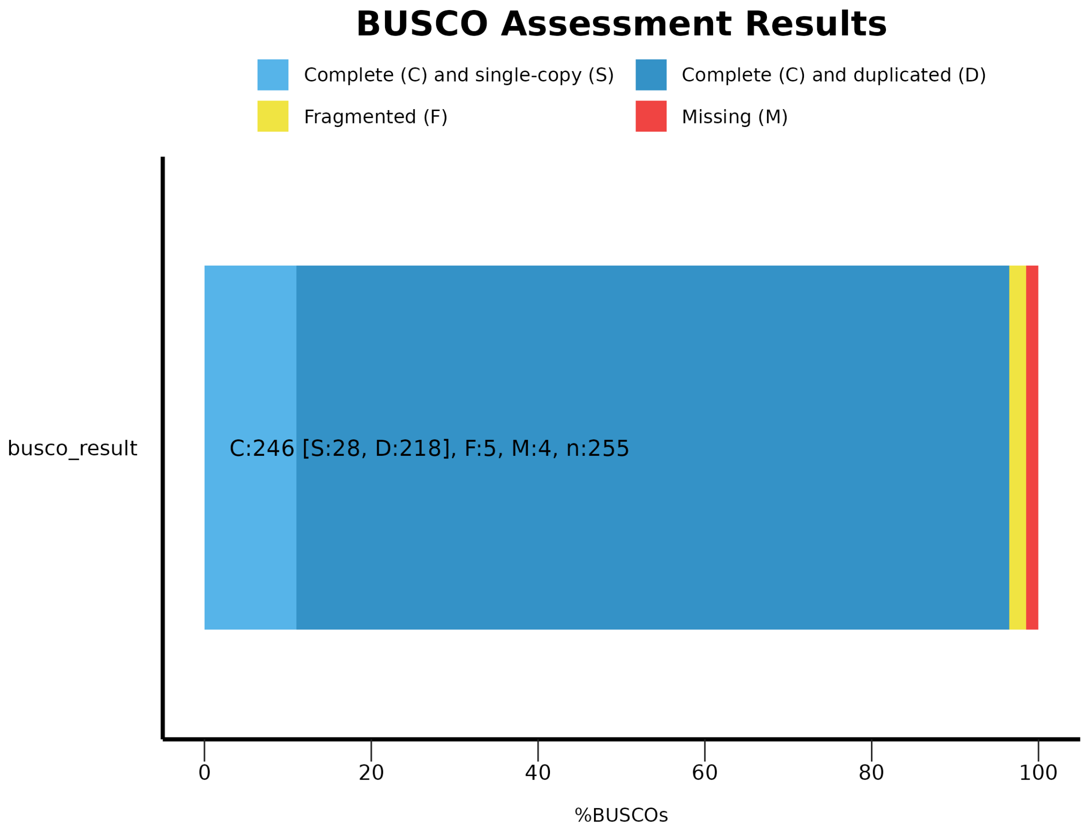

Figure S1. BUSCO completeness chart

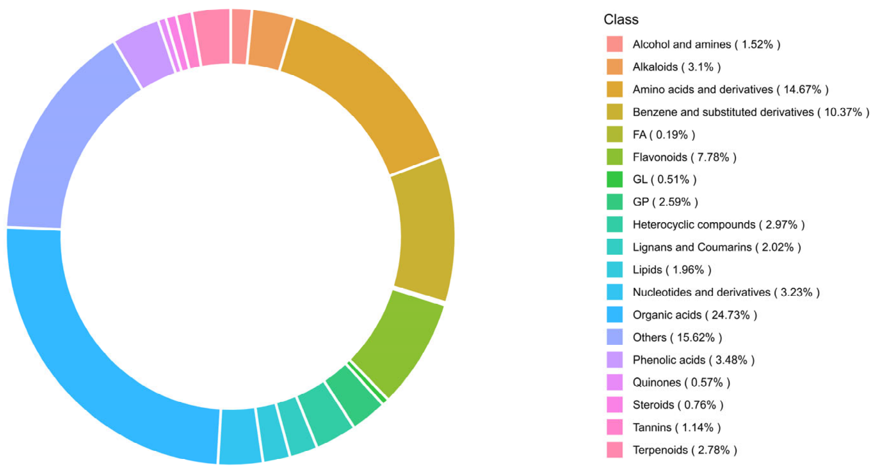

Figure S2. Classification diagram of metabolites

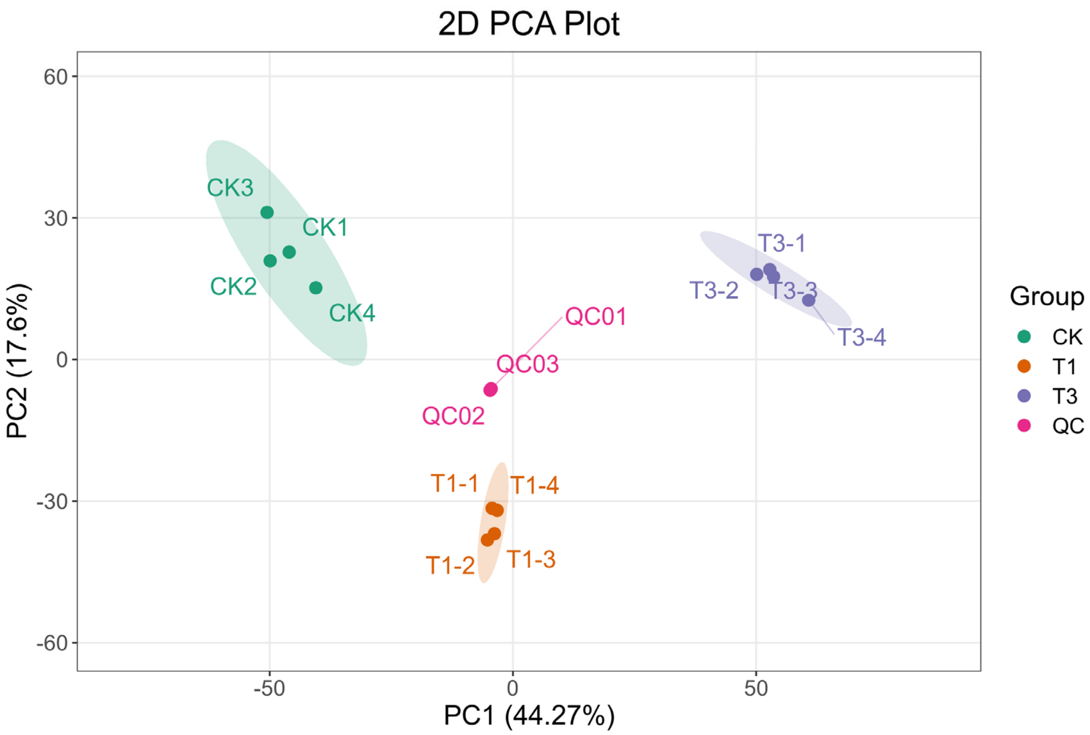

Figure S3. PCA score plot of metabolites

Table S1. Sequencing results of different treatments

Sample	Raw Reads	Raw Base(G)	Clean Reads	Clean Base(G)	Q20(%)	Q30(%)	GC Content(%)
CK1	69654684	10.45	68944268	10.34	99.34	97.66	46.9
CK2	54279886	8.14	53750976	8.06	99.32	97.6	46.81
CK3	57138354	8.57	56503476	8.48	99.38	97.83	46.60
CK4	62727858	9.41	61590194	9.24	99.35	97.71	46.93
T1-1	59742638	8.96	58613896	8.79	99.34	97.69	46.50
T1-2	56501058	8.48	55611458	8.34	99.3	97.53	46.45
T1-3	52076198	7.81	51198326	7.68	99.33	97.63	46.56
T1-4	52014376	7.80	51181654	7.68	99.28	97.46	46.44
T3-1	63584896	9.54	61888044	9.28	99.48	98.2	46.84
T3-2	62466552	9.37	61390916	9.21	99.41	97.96	46.96
T3-3	60768272	9.12	59397968	8.91	99.38	97.82	46.95
T3-4	52623874	7.89	51881160	7.78	99.32	97.61	46.62

Table S2. Statistical table of assembly results

Type	Number Mean Length	N50	N90	Type
Transcript	127986	1672	2597	836
Unigene	80072	2001	2844	1000

Table S3. Statistical table comparing clean reads of each sample to transcripts

Sample	Total Read pairs	Total mapped reads	Uniq mapped reads	Multiple mapped reads
CK1	34,472,134	32,213,730(93.45%)	8,766,044(25.43%)	23,447,686(68.02%)
CK2	26,875,488	25,092,031(93.36%)	6,740,144(25.08%)	18,351,887(68.28%)
CK3	28,251,738	26,381,447(93.38%)	7,006,300(24.80%)	19,375,147(68.58%)
CK4	30,795,097	28,714,217(93.24%)	7,656,292(24.86%)	21,057,925(68.38%)
T1-1	29,306,948	27,403,055(93.50%)	6,974,196(23.80%)	20,428,859(69.71%)
T1-2	27,805,729	25,824,785(92.88%)	6,542,982(23.53%)	19,281,803(69.34%)
T1-3	25,599,163	23,899,135(93.36%)	6,305,234(24.63%)	17,593,901(68.73%)
T1-4	25,590,827	23,862,907(93.25%)	6,199,812(24.23%)	17,663,095(69.02%)
T3-1	30,944,022	29,222,427(94.44%)	7,711,569(24.92%)	21,510,858(69.52%)
T3-2	30,695,458	28,766,570(93.72%)	7,592,995(24.74%)	21,173,575(68.98%)
T3-3	29,698,984	27,756,883(93.46%)	7,417,291(24.97%)	20,339,592(68.49%)
T3-4	25,940,580	24,212,588(93.34%)	6,516,809(25.12%)	17,695,779(68.22%)

Table S4. Annotations of differentially expressed genes in different databases

Database	Number of Genes Percentage	(%)
KEGG	39732	49.62
Nr	59642	74.49
SwissProt	47541	59.37
TrEMBL	59364	74.14
KOG	38563	48.16
GO	53168	66.40
Pfam	46175	57.67

Table S5.Top 20 differential metabolites between T1_vs_CK

Number	Index	Compounds	Log2FC	Type
1	MW0168257	2,3-Dimercaptosuccinic acid	6.24	up
2	MW0151580	Ile-Ser-Met	6.21	up
3	MW0151639	Ile-Val-Asn	5.57	up
4	MW0152921	Lys-Cys-Cys	5.46	up
5	MW0155826	Pro-Gln-Ala	5.17	up
6	MEDTN02215	Adenosine-5'-triphosphate	4.91	up
7	MW0145271	Arg-Asn-Gln-Glu	4.88	up
8	MW0120926	Anhydroleucovorin	4.86	up
9	MEDP2164	2,2-Dihydroxymethyl-1-azabicyclo[2.2.2]octan-3-one	4.85	up
10	MW0144664	Ala-Lys-His-Asp	4.75	up
11	MW0167804	[2-hexadecanoyloxy-3-[hydroxy-[2-(methylamino)ethoxy]phosphoryl]oxypropyl] (Z)-octadec-9-enoate	-5.30	down
12	MW0128729	[4-({[5,7-dihydroxy-2-(3,4,5-trihydroxyphenyl)-3,4-dihydro-2H-1-benzopyran-3-yl]oxy}carbonyl)-2,6-dihydroxyphenyl]oxidanesulfonic acid	-5.15	down
13	MW0150314	Gly-Asn-Leu-Arg-Lys	-4.44	down
14	MEDN1057	2-Methyl-d-erythritol 2,4-cyclodiphosphate	-4.42	down
15	MW0005906	1,5-Anthracenedisulfonic acid, 9,10-dihydro-9,10-dioxo-	-4.11	down
16	MW0001488	(2-Hydroxy-12-methoxy-18-methyl-11,17-dioxo-5-oxa-13,14,15,16-tetrathia-10,18-diazatetracyclo[10.4.2.01,10.03,9]octadeca-3,6-dien-8-yl) 3-(5-formyl-2-hydroxyphenoxy)-4-methoxybenzoate	-3.77	down
17	MW0159086	Val-Lys-Asp	-3.52	down
18	MW0128625	[2-hydroxy-6-(methoxymethyl)-3-(3,5,7-trihydroxy-3,4-dihydro-2H-1-benzopyran-2-yl)phenyl]oxidanesulfonic acid	-3.50	down
19	Lmdp004967	Kaempferol-3-O-(2''-feruloyl)glucosyl-(1→2)-(6''-malonyl)glucoside	-3.49	down
20	MW0162490	Bis(2,3-dihydroxypropyl) phosphate	-3.32	down

Number	Index	Compounds	Log2FC	Type
1	MW0168257	2,3-Dimercaptosuccinic acid	7.57	up
2	MEDN0553	2-Methylbenzoic acid	6.64	up
3	MW0152921	Lys-Cys-Cys	6.56	up
4	MW0158524	Tyr-Leu-Phe	6.36	up
5	MEDN0422	2'-Deoxyadenosine	6.34	up
6	MW0128423	[(1-{2,4-dihydroxy-5-[(2E)-4-hydroxy-3-methylbut-2-en-1-yl]phenyl}-3-[4-hydroxy-3-(3-methylbut-2-en-1-yl) phenyl]-1-oxopropan-2-yl)oxy]sulfonic acid	5.98	up
7	MEDTN0221 5	Adenosine-5'-triphosphate	5.94	up
8	MW0155392	Phe-Tyr-Ser	5.57	up
9	MW0109341	Pro-Tyr-Lys	5.54	up
10	MW0056798	PC(14:0/20:4(5Z,8Z,11Z,14Z))	5.45	up
11	MW0128205	(4-{6-[3,7-dihydroxy-2-(3-hydroxyphenyl)-3,4-dihydro-2H-1-benzopyran-4-yl]-2-(3,4-dihydroxyphenyl)-3,5,7 -trihydroxy-3,4-dihydro-2H-1-benzopyran-8-yl}-7-hydroxy-2-(3-hydroxyphenyl)-3,4-dihydro-2H-1-benzopyr an-3-yl)oxidanesulfonic acid	-8.53	down
12	MW0129595	1-(4-hydroxy-3-methoxyphenyl)-3-(4-methoxy-1-benzofuran-5-yl)propane-1,3-dione	-7.55	down
13	MEDL02568	FAA(17:1)	-7.22	down
14	MEDL01790	Cinnamtannin B1	-7.13	down
15	MW0148737	dTDP-4-amino-2,4-dideoxy-beta-L-xylose	-6.93	down
16	MW0109185	Phe-Met	-6.57	down
17	MW0011913	1,2-Dipalmitoleoyl-sn-glycero-3-phosphocholine	-6.27	down
18	Lmdp004967	Kaempferol-3-O-(2''-feruloyl)glucosyl-(1→2)-(6''-malonyl)glucoside	-6.16	down
19	MW0114263	Digalacturonate	-6.08	down

20	MW0106308	Cystathionine	-6.03	down

Table S6. Top 20 differential metabolites between T3_vs_CK

Table S7. Top 20 differential metabolites between T3_vs_T1

Number	Index	Compounds	Log2FC	Type
1	MW0152268	Leu-Asp-Pro-Asp-Tyr	5.22	up
2	MW0150993	His-Leu-Ile-Arg	5.09	up
3	MW0158524	Tyr-Leu-Phe	4.42	up
4	MW0150330	Gly-Asp-Ser	4.16	up
5	MW0158074	Tripteroside	3.89	up
6	ZINC13307982	Acumitin	3.86	up
7	MW0158504	Tyr-Ile-Met	3.79	up
8	MEDP1475	N-Methylhydantoin	3.70	up
9	MEDN0553	2-Methylbenzoic acid	3.56	up
10	MW0150095	Glu-Met-Val	3.53	up
11	MEDL02568	FAA(17:1)	-7.39	down
12	MW0128205	(4-{6-[3,7-dihydroxy-2-(3-hydroxyphenyl)-3,4-dihydro-2H-1-benzopyran-4-yl]-2-(3,4-dihydroxyphenyl)-3,5,7-trihydroxy-3,4-dihydro-2H-1-benzopyran-8-yl}-7-hydroxy-2-(3-hydroxyphenyl)-3,4-dihydro-2H-1-benzopyran-3-yl)oxidanesulfonic acid	-6.52	down
13	MW0129595	1-(4-hydroxy-3-methoxyphenyl)-3-(4-methoxy-1-benzofuran-5-yl)propane-1,3-dione	-6.15	down
14	MEDL01790	Cinnamtannin B1	-6.13	down
15	MW0148737	dTDP-4-amino-2,4-dideoxy-beta-L-xylose	-6.12	down
16	MW0144664	Ala-Lys-His-Asp	-5.99	down
17	MW0161605	(Z)-dehydrocyclopeptine	-5.97	down
18	MW0132836	3-[(1E)-4-hydroxybut-1-en-1-yl]-1H-isochromen-1-one	-5.23	down
19	MW0115046	O-6-deoxy-a-L-galactopyranosyl-(1->2)-O-b-D-galactopyranosyl-(1->3)-2-(acetylamino)-1,5-anhydro-2-deoxy-D-arabino-Hex-1-enitol	-5.18	down
20	MW0111404	(2E)-3-[4-({2-O-[(2S,3R,4R)-3,4-Dihydroxy-4-(hydroxymethyl)tetrahydro-2-furanyl]-beta-D-glucopyranosyl}oxy)-3-methoxyphenyl]acrylic acid	-5.17	down

Table S8. Top 20 flavonoid differential metabolites between T1_vs_CK

Number	Index	Compounds	Log2FC	Type
1	MW0137916	Delphinidin-3-o-arabinoside	4.40	up
2	MW0138292	Glycitin	3.65	up
3	MW0138602	Kaempferol 3-(2''-rhamnosyl-6''-acetylgalactoside) 7-rhamnoside	3.60	up
4	MW0127953	4-Hydroxychalcone	3.35	up
5	MW0139540	2-(3,4-dihydroxyphenyl)-5-hydroxy-3,7-bis[[(2S,3R,4R,5R,6S)-3,4,5-trihydroxy-6-methyloxan-2-yl]oxy]chromen-4-one	3.26	up
6	MW0138677	kaempferol 3-O-beta-D-glucopyranosyl-7-O-alpha-L-rhamnopyranoside	3.19	up
7	MW0139541	3-[(2S,3R,4S,5S,6R)-4,5-dihydroxy-6-(hydroxymethyl)-3-[(2S,3R,4R,5R,6S)-3,4,5-trihydroxy-6-methyloxan-2-yl]oxyoxan-2-yl]oxy-2-(3,4-dihydroxyphenyl)-5-hydroxy-7-[(2S,3R,4R,5R,6S)-3,4,5-trihydroxy-6-methyloxan-2-yl]oxychromen-4-one	2.94	up
8	MW0139746	Spinosin	2.85	up
9	MEDL02159	2'-Hydroxydaidzein	2.73	up
10	MWS-00-245g_N	Rebaudioside A	2.57	up
11	Lmdp004967	Kaempferol-3-O-(2''-feruloyl)glucosyl-(1→2)-(6''-malonyl)glucoside	-3.49	down
12	FDATN00279	Morin hydrate	-2.93	down
13	MW0138770	Licochalcone a	-2.11	down
14	MW0138377	Hesperidin	-2.00	down
15	MW0158074	Tripteroside	-1.65	down
16	MW0113884	Aloeresin A	-1.63	down
17	MW0139473	Quercetagitrin	-1.59	down
18	MW0169603	Osajin	-1.53	down
19	MW0124445	Irisxanthone	-1.51	down

20	MW0148229	delphinidin 3-O-(6''-O-malonyl)-beta-D-glucoside-3'-O-beta-D-glucoside	-1.45	down

Table S9. Top 20 flavonoid differential metabolites between T3_vs_CK

Number	Index	Compounds	Log2FC	Type
1	MW0139490	[6-[2-(3,4-Dihydroxyphenyl)-5,7-dihydroxy-4-oxochromen-3-yl]oxy-3,4,5-trihydroxyoxan-2-yl]methyl acetate	4.34	up
2	MW0139540	2-(3,4-dihydroxyphenyl)-5-hydroxy-3,7-bis[[(2S,3R,4R,5R,6S)-3,4,5-trihydroxy-6-methyloxan-2-yl]oxy]chromen-4-one	4.29	up
3	MW0139541	3-[(2S,3R,4S,5S,6R)-4,5-dihydroxy-6-(hydroxymethyl)-3-[(2S,3R,4R,5R,6S)-3,4,5-trihydroxy-6-methyloxan-2-yl]oxyoxan-2-yl]oxy-2-(3,4-dihydroxyphenyl)-5-hydroxy-7-[(2S,3R,4R,5R,6S)-3,4,5-trihydroxy-6-methyloxan-2-yl]oxychromen-4-one	4.27	up
4	MW0138677	kaempferol 3-O-beta-D-glucopyranosyl-7-O-alpha-L-rhamnopyranoside	4.05	up
5	MW0137916	Delphinidin-3-o-arabinoside	3.98	up
6	MW0138292	Glycitin	3.94	up
7	MWS-00-245g_N	Rebaudioside A	3.81	up
8	MW0139304	Peonidin-3-O-alpha-arabinopyranoside	3.61	up
9	MW0139746	Spinosin	3.23	up
10	MW0138602	Kaempferol 3-(2''-rhamnosyl-6''-acetylgalactoside) 7-rhamnoside	3.07	up
11	Lmdp004967	Kaempferol-3-O-(2''-feruloyl)glucosyl-(1→2)-(6''-malonyl)glucoside	-6.16	down
12	MW0138377	Hesperidin	-5.74	down
13	MW0113884	Aloeresin A	-5.70	down
14	mws0895	Genistin	-5.48	down
15	MW0148229	delphinidin 3-O-(6''-O-malonyl)-beta-D-glucoside-3'-O-beta-D-glucoside	-5.43	down
16	MW0138082	Epigallocatechin-(4beta->8)-epicatechin-3-O-gallate ester	-5.33	down
17	MW0139516	Quercetin 3-O-(6''-acetyl-glucoside)	-5.27	down
18	MW0138673	2''-p-Coumarylastragalin	-4.84	down
19	MW0139530	Quercetin 3-xylosyl-(1->6)-glucoside	-4.82	down

20	MW0137590	Cardamonin	-4.70	down

Table S10. Top 20 flavonoid differential metabolites between T3_vs_T1

Number	Index	Compounds	Log2FC	Type
1	MW0158074	Tripteroside	3.89	up
2	MW0139490	[6-[2-(3,4-Dihydroxyphenyl)-5,7-dihydroxy-4-oxochromen-3-yl]oxy-3,4,5-trihydroxyoxan-2-yl]methyl acetate	3.50	up
3	MW0138459	Isobavachalcone	2.00	up
4	MW0139304	Peonidin-3-O-alpha-arabinopyranoside	1.77	up
5	MW0117142	1-Hydroxy-3,6,7-trimethoxy-2-(2-hydroxy-3-methyl-3-butenyl)-8-(3-methyl-2-butenyl)-xanthone	1.66	up
6	MW0122798	alpha-Mangostin	1.58	up
7	MW0138534	Dactilin	1.53	up
8	MW0139541	3-[(2S,3R,4S,5S,6R)-4,5-dihydroxy-6-(hydroxymethyl)-3-[(2S,3R,4R,5R,6S)-3,4,5-trihydroxy-6-methyloxan-2-yl]oxyoxan-2-yl]oxy-2-(3,4-dihydroxyphenyl)-5-hydroxy-7-[(2S,3R,4R,5R,6S)-3,4,5-trihydroxy-6-methyloxan-2-yl]oxychromen-4-one	1.33	up
9	MEDL02035	Myricetin	1.32	up
10	MWS-00-245g_N	Rebaudioside A	1.25	up
11	mws0895	Genistin	-4.51	down
12	MW0138082	Epigallocatechin-(4beta->8)-epicatechin-3-O-gallate ester	-4.26	down
13	MW0113884	Aloeresin A	-4.07	down
14	MW0139516	Quercetin 3-O-(6"-acetyl-glucoside)	-4.07	down
15	MW0148229	delphinidin 3-O-(6"-O-malonyl)-beta-D-glucoside-3'-O-beta-D-glucoside	-3.99	down
16	MW0138673	2"-p-Coumarylastragalin	-3.96	down
17	MW0139530	Quercetin 3-xylosyl-(1->6)-glucoside	-3.96	down
18	MEDL01953	quercetin 3-O-glucuronide	-3.74	down
19	MW0138377	Hesperidin	-3.74	down
20	MW0130165	5,7,3',4'-Tetrahydroxy-6,8-dimethoxyflavone	-3.68	down
